# Effects of freshwater blue spaces may be beneficial for mental health: A first, ecological study in the North American Great Lakes region

**DOI:** 10.1371/journal.pone.0221977

**Published:** 2019-08-30

**Authors:** Amber L. Pearson, Ashton Shortridge, Paul L. Delamater, Teresa H. Horton, Kyla Dahlin, Amanda Rzotkiewicz, Michael J. Marchiori

**Affiliations:** 1 Michigan State University, Department of Geography, Environment & Spatial Sciences, East Lansing, MI, United States of America; 2 University of Otago, Department of Public Health, Wellington, New Zealand; 3 University of North Carolina at Chapel Hill, Department of Geography and Carolina Population Center, Chapel Hill, NC, United States of America; 4 Northwestern University, Department of Anthropology, Evanston, IL, United States of America; Tanzania Fisheries Research Institute, UNITED REPUBLIC OF TANZANIA

## Abstract

Research linking green space and mental health abounds. It also appears that oceanic blue spaces may be salutogenic, benefitting mental health through their expansive viewscapes, and possibly auditory and olfactory stimuli. Yet, it is unknown whether the same is true for freshwater bodies. In this ecological study, we explored associations between hospitalizations for anxiety/mood disorder in Michigan (>30,000) and proximity to the North American Great Lakes. As a sensitivity analysis, we examined associations for 15 different inland lake sizes. Results showed small, protective effects for distance to Great Lake (β = 0.06, *p*<0.001) and percentage of inland lakes (β = -0.04, *p* = 0.004). Unexpectedly, shorter distance to nearest inland lake was associated with higher anxiety/mood disorder hospitalizations. The protective effects of percentage area covered by inland lakes was observed for all lake sizes. These initial findings provide a foundation for future individual-level research with finer measurement of health outcomes and blue space exposure.

## Introduction

People’s environmental surroundings contribute in profound but complex ways to their mental and physical health [1, 2]. In the past two decades, a growing body of research has focused on the role of proximity to green (e.g., parks) and blue (e.g., waterbodies) spaces in the promotion of mental health. While much published work has examined green space (e.g., [3–9]), the contribution of blue space to health is an emerging line of research and has been primarily limited to examination of oceanic blue space [10–15]. Causal pathways linking freshwater blue spaces to mental health are similar in several respects to oceanic blue spaces (particularly with respect to physical activity and social interactions) but a third pathway, focusing on direct therapeutic benefits, has not been articulated or evaluated on a broad scale for large and small freshwater blue spaces. In this large ecological study, we address this gap by linking hospitalizations associated with anxiety/mood disorder to exposure to large and small freshwater lakes while accounting for other factors.

Quantifying health effects of proximity and exposure to freshwater blue space is relatively understudied; a 2011 review found 36 papers on this topic [16]. Laboratory research suggests that blue hues may confer relaxation, whereby the hue in images of water have been significantly associated with aesthetic preference and naturalness [17] and perceived restorativeness [15]. Still, water is not always blue in color, and water may not always be imbued with positive meanings [18]. Other theoretical pathways through which blue spaces benefit physical health include ‘blue recreation’ that entails physical activity on beaches or shores, or swimming [14]. Proximity to coasts may increase accessibility of blue spaces, and has been positively associated with mental health [19].

The North American Great Lakes, which are visually similar to oceans, offer a unique opportunity to evaluate the generalizability of findings of health benefits from oceanic blue spaces. Likewise, the evaluation of exposure to small, inland lakes may help us understand the effects of smaller blue spaces.

In this study, possibly the first to integrate a large hospitalization dataset with geographic measures of proximity to multiple sizes of freshwater blue spaces, we provide descriptive summaries of their integration and evaluate two fundamental hypotheses:

1. Proximity to Great Lakes blue space is associated with lower hospitalization rates for anxiety and mood disorders, while adjusting for age, sex, area-level poverty, and population density.
2. Proximity to inland lakes blue space is associated with lower hospitalization rates for anxiety and mood disorders, while adjusting for age, sex, area-level poverty, and population density.

We also develop a sensitivity analysis to test for a possible threshold size at which inland lakes appear to influence mental health.

## Materials and methods

### Study area

**Fig 1:**
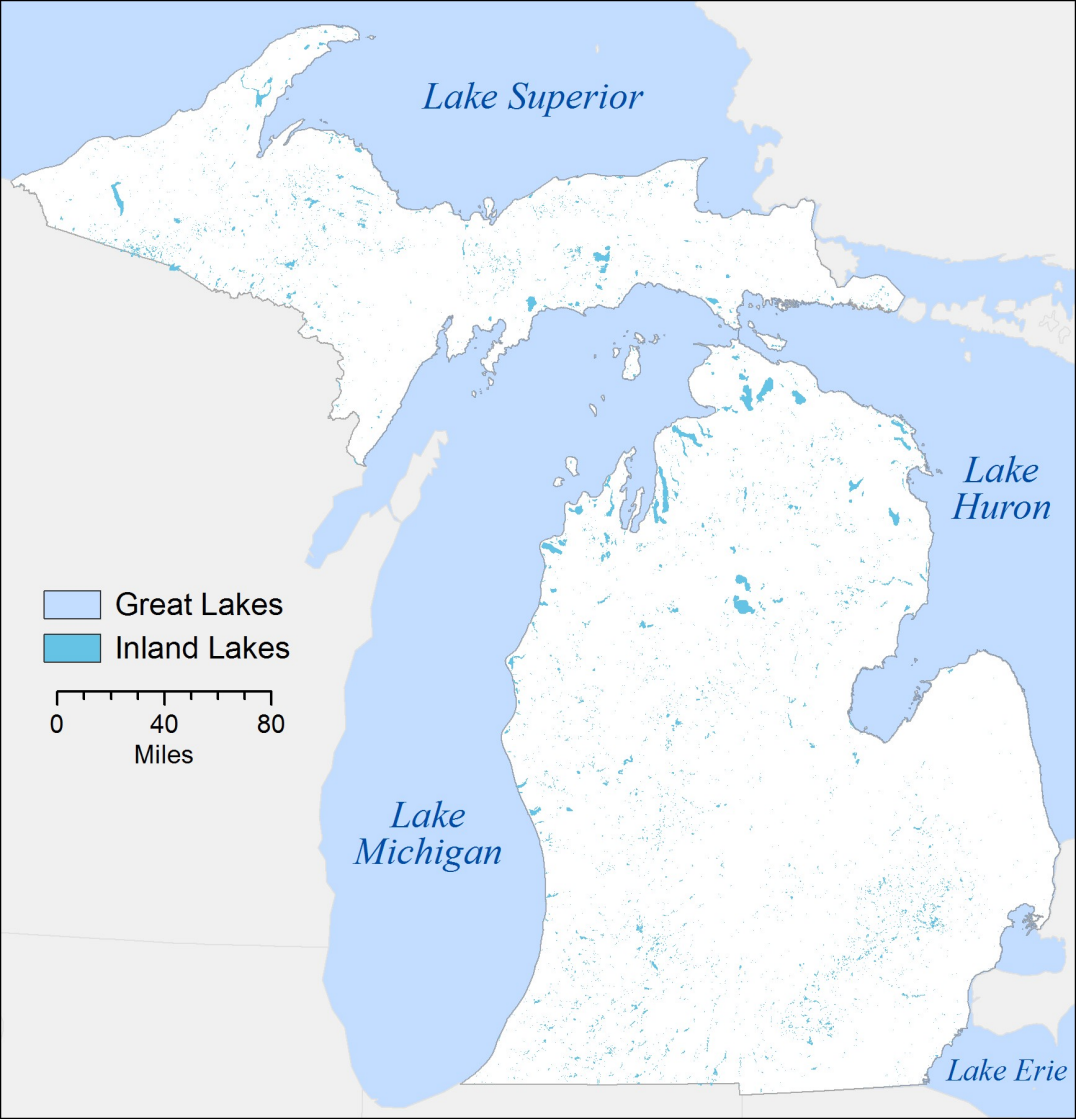
Map of Michigan’s inland lakes, surrounded by North American Great Lakes (created by authors).

The State of Michigan is comprised of two peninsulas, which are surrounded by four of the five North American Great Lakes (Fig 1). The state also hosts ~62,000 small inland lakes (mean area 0.09 km^2^) and a population of nearly 10 million people [20]. Lakes are permanent, given Michigan’s high and temporally consistent level of precipitation (820 mm/year). Seasonal water features such as vernal pools are excluded from this analysis.

### Data

Population and health information for this study, including general demographic and hospitalization data, were obtained from ZIP codes represented as US Census ZIP Code Tabulation Area (ZCTA) polygons (n = 915) covering Michigan. For each ZIP code, we compiled population counts for five-year age groups between 15 years to 85+ years, stratified by sex, using the 2010 census [20]. We also compiled median household income and calculated population density by dividing the total population by the land area, yielding a value of people per km^2^. All hospital admissions (in 2014) for those aged 15+ years for anxiety/mood disorder were extracted from the Michigan Inpatient Database (MIDB). Nearly all inpatient hospitals (97.7%) and most inpatient hospitals that offer psychiatric services (73.7%) report to the MIDB, but not all (see Statistical analyses for consideration of possible missing data due to reporting practices). The MIDB data were aggregated to counts of hospitalizations by five-year age-sex groups by ZIP code. Spatial data for the Great Lakes boundaries [21], Michigan inland lakes and road networks [22] were compiled in ArcMap 10.5 (ESRI, Redlands, CA).

### Ethical approval

Ethical exemption was provided as the secondary data used here were de-identified prior to receipt (IRB #07–362– April 2013).

### Quantification of blue space exposure measures

We calculated: 1) the proportion of a ZIP code occupied by inland lakes; and 2) the average Euclidean distance to the nearest blue space boundary, distinguished between inland lakes and Great Lakes. To calculate the proportion of a ZIP code occupied by inland lakes, we calculated the area occupied by inland lakes and divided it by the total area (km^2^) of each ZIP code. To calculate distance to blue space, we generated 30 random points along the road network (as most people reside along roads) in each ZIP code and measured the distance to the nearest inland lake and Great Lake separately; we then calculated the average of the 30 measurements for each ZIP code. This is a reasonable approximation for the majority population, as used in other research [23].

To conduct a sensitivity analysis based on possible difference in effects based on the size of the inland lake, we calculated both distance to and proportion of ZIP code occupied by blue space when restricting the data to a suite of minimum lake sizes ranging from 0.01 km^2^ up to 1 km^2^. This resulted in 15 variables each for percent and distance to inland lakes per ZIP code. All spatial calculations were conducted using ArcMap 10.5.

### Statistical analyses

We first calculated descriptive statistics for individual-level anxiety/mood disorder hospitalizations by sex. Then, we calculated descriptive statistics for ZIP code characteristics stratified by high/low occurrence of anxiety/mood disorder hospitalizations (using the median as a cutoff). Next, we tested for associations between the blue space exposure measures and frequency of anxiety/mood disorder hospitalizations by fitting negative binomial (due to over-dispersion in the data) regression models. These models had age-sex specific counts of anxiety/mood disorder as the dependent variable, age-sex population counts as an offset, blue space exposures (for each measure, separately) as the independent variable of interest, and were adjusted for median income and population density.

Due to the incomplete nature of our MIDB hospitalization data, we also conducted a post-hoc sensitivity analysis (identical to the regression modeling outlined above) on a subset of ZIP codes (n = 284) that either contain a psychiatric hospital that reports to the MIDB or are located within 15 miles of one (Euclidean distance measured from the population-weighted centroid of the ZIP code). We observed similar trends to those reported here (results available upon request).

All statistical analyses were conducted using Stata v14 (College Station, TX). Standardized β coefficients are reported for comparisons across models.

## Results

There were >30,000 anxiety/mood disorder hospitalizations in Michigan in 2014; most of the people hospitalized were white (84%) (Table 1). On average, patients were 42 years old. The lowest proportions of hospitalizations were observed on weekend days (10–11%) and in the month of February (7%). Similar trends were observed for males and females. The average distance to a Great Lake was slightly lower for ZIP codes without any anxiety/mood disorder hospitalizations (mean = 34km, sd = 28km) (Table 2). There was no difference in average distance to nearest inland lake by level of hospitalizations. However, there was a somewhat larger percentage of the ZIP code covered in inland lakes for the group without any hospitalizations (mean = 3%, sd = 6%) compared to those with hospitalizations (mean = 2%, sd = 4–5%).

**Table 1:** Descriptive statistics of individuals hospitalized for anxiety or mood disorders in Michigan, 2014.

Characteristics	Male	Female	Total
***n***	*13903*	*16518*	*30421*
***Demographics***			
Age, mean (sd)	41 (16)	44 (17)	42 (16)
Ethnicity, n (%)			
White	10845 (85)	12669 (84)	23514 (84)
Black	1664 (13)	2123 (14)	3787 (14)
Other	290 (2)	375 (2)	665 (2)
Day of the week, n (%)			
Sunday	1407 (10)	1640 (10)	3047 (10)
Monday	2207 (16)	2617 (16)	4824 (16)
Tuesday	2316 (17)	2782 (16)	5098 (17)
Wednesday	2135 (15)	2559 (16)	4694 (15)
Thursday	2134 (15)	2641 (16)	4775 (16)
Friday	2146 (16)	2535 (15)	4681 (15)
Saturday	1558 (11)	1744 (11)	3302 (11)
Month, n (%)			
January	1081 (8)	1349 (8)	2430 (9)
February	1004 (7)	1250 (8)	2254 (7)
March	1169 (8)	1317 (8)	2486 (8)
April	1141 (8)	1371 (8)	2512 (8)
May	1154 (8)	1440 (9)	2594 (9)
June	1054 (8)	1406 (8)	2460 (8)
July	1224 (9)	1430 (9)	2654 (9)
August	1315 (9)	1494 (9)	2809 (9)
September	1198 (9)	1474 (9)	2672 (9)
October	1211 (9)	1445 (9)	2656 (9)
November	1127 (8)	1296 (8)	2423 (8)
December	1225 (9)	1256 (7)	2481 (8)

**Table 2:** Characteristics of zip codes, stratified by levels of anxiety/mood disorder hospitalizations.

Characteristics	No hospitalizations	Low Rate (1–554)	High Rate (545+)	All
***Zip code land area*, *mean (sd)***				
Land area in km^2^	165 (169)	151 (167)	172 (217)	165 (199)
***Distance to water bodies*, *mean (sd)***				
Average distance to Great Lake in km	34 (28)	35 (27)	35 (32)	35 (30)
Average distance to inland lake in km	1 (1)	1 (1)	1 (1)	1 (1)
***Water body coverage of zip code*, *mean (sd)***				
Percent inland lake	3 (6)	2 (4)	2 (5)	2 (5)

**Fig 2:**
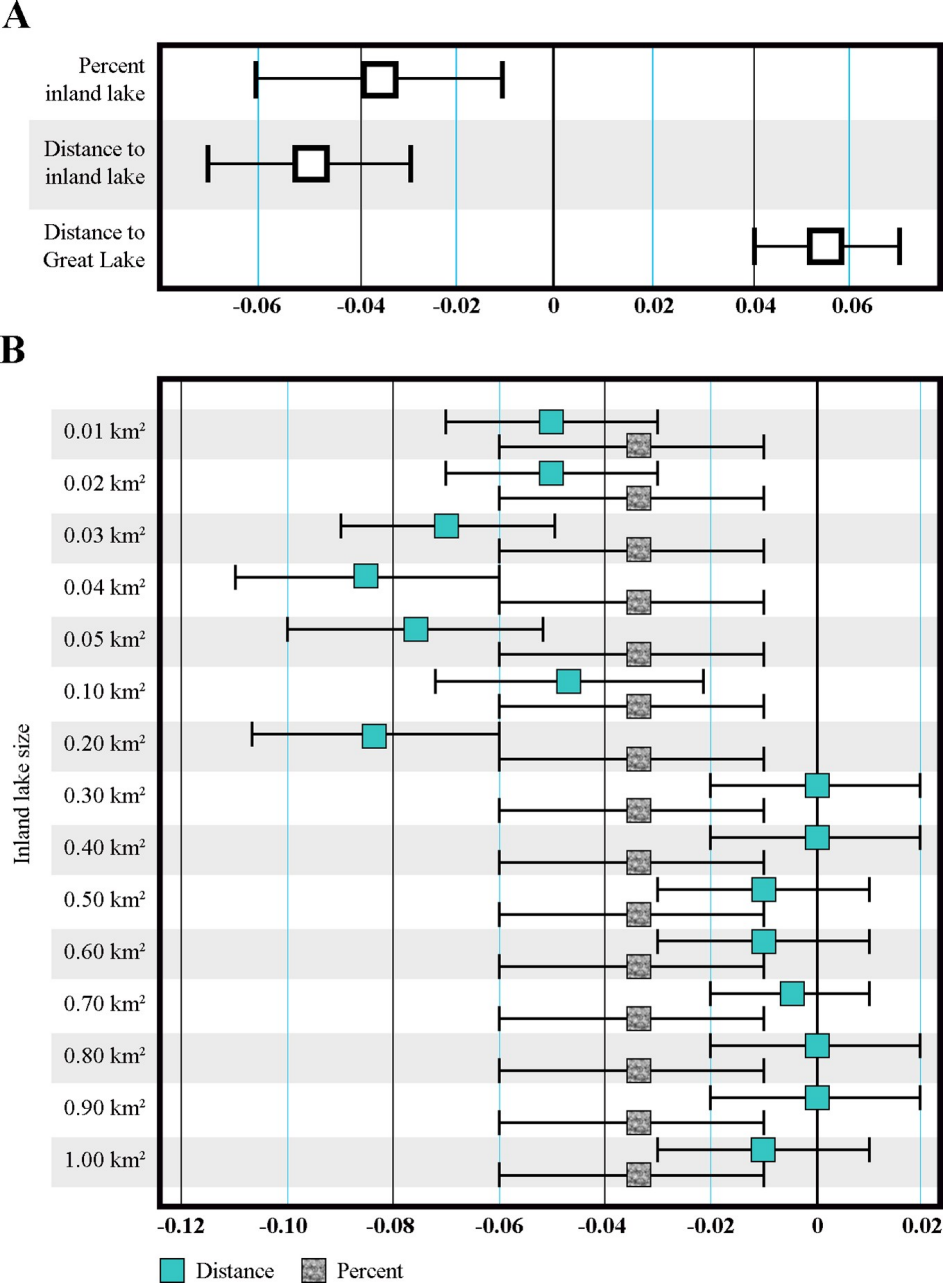
Regression modelling results predicting anxiety/mood disorder hospitalizations. (A) Primary analyses and (B) Sensitivity Analyses based on inland lake size.

From our primary independent variables of interest, both percentage of inland lakes and distance to Great Lake showed protective associations with anxiety/mood disorder hospitalizations, after adjustment for population density, age, sex and median household income (Fig 2A). Greater area occupied by inland lakes was associated with a lower anxiety/mood disorder hospitalization rate (standardized β = -0.04, *p* = 0.004). The average predicted count of anxiety/mood disorder hospitalization would be 1.2 when percent inland lake is zero. When percent inland lake increases to 40%, the average predicted count would be 0.86.

A shorter distance to a Great Lake was associated with lower anxiety/mood disorder hospitalization rates (standardized β = 0.06, *p* < 0.001). When the distance to the nearest Great Lake is 100 km, the average predicted count would be 1.3. Unexpectedly, shorter distance to nearest inland lake was associated with higher anxiety/mood disorder hospitalization rates (standardized β = -0.05, *p* < 0.001). This trend was consistent in the sensitivity analysis (Fig 2B) such that shorter distances were associated with higher hospitalizations, from lake sizes 0.01 km^2^ to 0.20 km^2^ (roughly 0.08 mi^2^). The effect attenuated for larger inland lakes. Also in sensitivity analyses, the protective effects of percentage area covered by inland lakes were observed for all sizes. From all models, the largest protective effect was observed for distance to Great Lake. Still, all analyses showed relatively small effect sizes.

## Discussion and conclusion

We observed conflicting findings in relation to distance to blue space for Great Lakes versus inland lakes. We found a protective effect for Great Lakes, whereby living closer to a Great Lake was associated with lower anxiety/mood disorder hospitalizations. In contrast, we observed that living closer to an inland lake was associated with higher hospitalizations. This was particularly true for the smallest inland lakes and the effect attenuated for larger inland lakes. The conflicting results suggest that freshwater may, in fact, have small salutogenic effects but only for larger water bodies. It is possible that these differences relate to the expansiveness of the view, or the auditory environment associated with larger water bodies–but this could not be directly tested in our study. Our unexpected finding that shortened distances to small inland lakes was associated with increased anxiety/mood disorder warrants consideration. First, Michigan’s inland lakes are commonly managed by Property Owner’s Associations (POA), in which residents of the lake are responsible for determining waterfront accessibility, organizing social events, and coordinating with the Department of Natural Resources to protect the lake environment [24]. Consequently, lake access may be restricted to members of the POA. For example, even with >60,000 inland lakes, Michigan has only 1,800 public boat launches and only 30% of the shoreline is open to the public [22, 25]. It is also possible that those who live near small lakes are more likely and able (financially and/or socially) to seek medical treatment for anxiety/mood disorders. A study from the Netherlands, similarly found that nearby blue space (oceanic) was associated with lower perceived general health, whilst water sources 1-3km were associated with better perceived health [26]. Other research from New Zealand found an association between visible ocean and psychological distress and, in this setting, blue space was almost always distant (>3km) [5]. A wider exploration of mental health outcomes and individual-level measurement could clarify this finding. In addition, these effects may differ by age [27], sex, and other characteristics as previous research has shown that the importance of nature for those visiting freshwater blue spaces varies by sociodemographics and age [28].

We also found that a higher proportion of inland lake area was associated with lower anxiety/mood disorder hospitalizations. Since we also found that larger lakes were associated with lower hospitalizations, it is possible that abundance of blue space may be important to yield benefits as echoed in our finding that proximity to a Great Lake was protective. It is also possible that having multiple, nearby lakes creates a habitat or migration corridor for certain species of birds or other wildlife that also contribute to the aesthetic or restorative dimensions of blue space.

While this study appears to be the first to consider mental health-freshwater associations at a regional scale, important limitations must be considered. This study is limited in both the scale and the severity of the health outcome, as hospitalizations were the only state-wide data available and are only available at the ZIP code level. The nature of the relationship of blue space and mental health may require assessment of exposures (and outcomes) at a finer spatial resolution (individual) than is achievable from ZIP code aggregation. As an ecological study, findings cannot be generalized to individuals. Additionally, the incorporation of measures for less severe illnesses or indicators of stress may be beneficial. In particular, subclinical measures may be more appropriate to understand the health benefits of blue spaces, especially when investigating proximity of residence to an inland lake. Further, this was an ecological study and thus could not adjust for individual covariates that may be important in the blue and green space-mental health pathway including ethnicity, wealth, other causes of stress, time spent outdoors and health-seeking behaviors. We also did not account for differences in the availability of hospitals and other community mental health centers for treatment. The study area includes most of the North American Great Lakes and thousands of inland lakes. It is unclear whether the effects observed in this study would be found in drier settings with far fewer freshwater bodies. While future investigations could optimally measure longitudinal and individual-level visual and auditory blue space exposures and mental health outcomes, this study provides an initial foundation for such research on whether and at what scale freshwater blue spaces may confer mental health benefits.

In summary, this first ecological study of the potential mental health benefits of freshwater blue spaces in the North American Great Lakes region showed small but protective effects of proximity to a Great Lake and abundance of inland lakes. In contrast, proximity to an inland lake exhibited negative effects which attenuated as lake size increased. Taken together, these findings suggest that larger freshwater and abundant blue spaces may be more likely to confer benefits. Future research in a variety of settings could usefully confirm these findings.

## Supporting information

S1 File Dataset used for analyses in this manuscript. (CSV)
